# Investigation on Mechanical Performance and Flame-Retardancy of Polymer Cement-Based Coatings with Ettringite Modified by Amphiphilic Group

**DOI:** 10.3390/polym18070863

**Published:** 2026-03-31

**Authors:** Fangzhou Yin, Kai Ma, Xingyu Gan, Laibo Li, Haiming Zhang, Lingchao Lu

**Affiliations:** Shandong Provincial Key Laboratory of Green and Intelligent Building Materials, University of Jinan, Jinan 250022, China

**Keywords:** polymer, cement-based coatings, flame retardancy, ettringite

## Abstract

Polymer cement-based coatings (PCC) have great potential in protecting underground structures, while their flame -retardancy is limited by the flammability of the polymer components. Developing multifunctional inorganic fillers that can enhance both mechanical properties and flame retardancy is crucial for improving the safety and durability of the coatings. In this study, an ettringite (AFt) modified by amphiphilic group and aluminum hydroxide (AH) was subjected to surface functionalization treatment and then incorporated into the PCC to enhance the overall performance of the material. The tensile strength and bond strength of the coatings modified by amphiphilic group with AFt were increased by 27.7% and 22.4%, respectively. Meanwhile, the flame burn time, flameless burn time, and carbonization length were reduced by 89.1%, 80.6%, and 90.2%, respectively, and the limiting oxygen index (LOI) increased by 28.9%. The amphiphilic groups act as molecular bridges that couple the inorganic modified AFt with the organic VAE phase, thereby strengthening the organic–inorganic interface and promoting a more integrated polymer–cement network. Meanwhile, the well-dispersed inorganic phases provide endothermic dehydration and a protective residue during heating, jointly improving the mechanical reliability and fire safety of the PCC.

## 1. Introduction

In modern underground engineering, structural safety has always been a crucial issue, as complex geological conditions and restricted working environments often lead to dangerous situations such as tunnel collapses and water seepage [1]. Therefore, sealing materials are widely used to repair defects, prevent leaks, and provide protective reinforcement for underground engineering structures [1,2]. The traditional sealing materials are divided into inorganic materials and organic materials. Inorganic sealing materials, such as shotcrete, offer high compressive strength, rapid setting, low technical demands for application, and wide raw-material availability [3,4,5,6]. However, shotcrete has notable drawbacks in practice: its thick coating results in high material consumption and rebound loss [6,7], while its inherent brittleness and low toughness make it prone to cracking and sealing failure under the micro-movements of underground roadways, posing considerable safety risks in coal mines [7]. Organic polymer coatings have been widely used for surface protection and repair due to excellent flexibility [8,9]. However, the application of these coatings remains limited by two major issues. First, these coatings are inherently flammable and may release large amounts of smoke and toxic gases during fire exposure, posing serious safety concerns in underground engineering [10,11]. Second, volatile organic compounds and irritating substances may be emitted during application and maintenance, potentially threatening the health of construction workers [4,12]. Therefore, polymer cement-based coatings, which integrate the advantages of both inorganic and organic materials, have attracted increasing research interest in recent years for underground sealing and protection applications [1,2].

The polymer cement-based coatings exhibit unique advantages in functional complementarity by combining the durability of inorganic materials with the ductility of organic polymers, and have become an important research focus with broad engineering application prospects [5,13]. However, the inherent flammability of organic polymer components can significantly reduce the overall fire resistance of coatings [14]. Adding flame retardancy into polymer cement-based coatings is an effective way to improve its flame retardant performance [11,15,16]. Feng et al. [17] reported that halogenated flame-retardants possess the outstanding advantages of high flame retardant efficiency and low required dosage. Li et al. [18] demonstrated that the incorporation of phosphorus-based flame retardants can significantly improve the flame-retardant performance of polymers, increasing the limiting oxygen index (LOI) to 29.2% while effectively suppressing heat release, smoke generation, and CO_2_ emission during combustion. Although these methods can effectively enhance the flame-retardant properties of polymers, the application of such approaches may still involve potential safety risks [19]. During the combustion process, they release a large amount of toxic and corrosive gases and smoke, which can cause serious harm to human health and the environment [20,21]. Therefore, there is an urgent need to propose a new type of flame retardant that should not only have efficient flame-retardant properties but also be non-toxic and environmentally friendly. To help readers better understand the differences between traditional halogenated flame retardants and emerging non-halogenated flame retardants, a comparative summary is presented in Table 1.

In recent years, inorganic flame retardants have become promising alternatives to traditional organic flame-retardant additives because of their low toxicity and high thermal stability [11]. Among these substances, AFt containing a large amount of chemically bound water has considerable application potential [22,23]. The flame-retardant effect of AFt mainly stems from the crystalline water it releases during the thermal decomposition process. This water can absorb heat and inhibit combustion [22,23]. However, the practical application of AFt as a flame retardant still has some limitations. The unmodified AFt particles are prone to agglomeration and have poor compatibility with the polymer matrix at the interface, resulting in uneven dispersion and weak interfacial bonds. Therefore, surface modification may improve the applicability of AFt in polymer cement-based coatings [24,25]. To address this issue, functionalization modification was carried out on AFt to enhance its dispersibility and interfacial interaction, providing a feasible approach to improve its flame retardancy.

The use of amphiphilic groups and AH to modify the surface of AFt was proposed in this study [26,27]. By introducing an amphiphilic group and AH as a molecular linker, the interfacial compatibility between the modified filler and the polymer matrix was significantly improved, together with enhanced filler dispersion [27,28,29]. Subsequently, this synthetic filler material was added to the composite coating material composed of polymer emulsion and cement. The systematic assessment was conducted on the mechanical properties, microstructure, and flame retardancy of the coatings.

## 2. Materials and Methods

### 2.1. Materials

The white portland cement (WPC) used in this study was 42.5-grade cement produced by Aalborg portland (Anqing) Co., Ltd. (Anqing, China), and its chemical composition was listed in Table 2. The heavy calcium carbonate (HC) and quartz powder (QP) were supplied by Shandong Hengfu Non-metallic Materials Co., Ltd. (Zibo, China). The calcium hydroxide (CH), aluminum sulfate octadecahydrate (AS), aluminum hydroxide (AH), sodium hydroxide (SH), and γ-aminopropyltriethoxysilane (amphiphilic group, AG) were purchased from Shanghai Aladdin Biochemical Technology Co., Ltd. (Shanghai, China). The mineral oil-based defoamer, vinyl acetate ethylene copolymer emulsion (VAE), and superplasticizer (SP) were supplied by Guangdong Tianfeng Co., Ltd. (Chaozhou, China).

### 2.2. Sample Preparation

#### 2.2.1. Preparation of Ettringite

**Figure 1:**
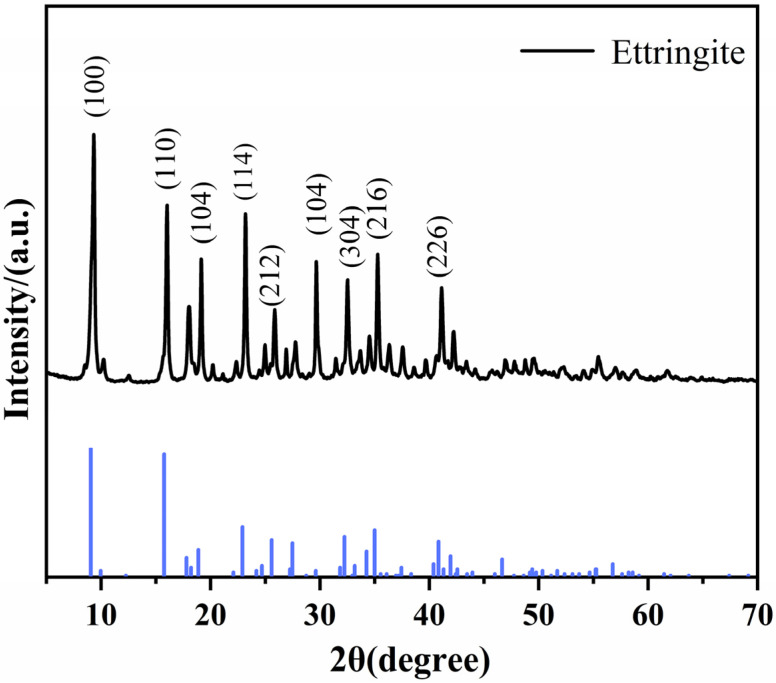
XRD patterns of AFt.

The deionized water and anhydrous ethanol were mixed at a volume ratio of 2:1 to prepare a mixed solvent. The CH was added to the above solvent and continuously stirred using a magnetic stirrer at 500 rpm for 30 min until complete dissolution was achieved, forming solution A. Meanwhile, AS and SH were dissolved under the same solvent system and stirring conditions (500 rpm for 30 min) to obtain solution B. Solution A was mixed with solution B, and then a continuous stirring reaction was conducted at 50 °C in a water bath at a speed of 300 r/min for 12 h. After the reaction was complete, the precipitate was collected by centrifugation at a speed of 10,000 rpm for 10 min at 15 °C and thoroughly washed three times with anhydrous ethanol to remove the residual unreacted AG solution. The collected solid was subjected to vacuum drying at 40 °C for 12 h, followed by grinding and sieving to obtain the AFt. Its XRD pattern was shown in Figure 1 [30]. The XRD pattern of the synthesized AFt was shown in Figure 1, where the diffraction peaks observed at 2θ values of 9.15°, 15.84°, 18.95°, 22.92°, 25.60°, and 34.99° were assigned to the characteristic crystal planes of ettringite (AFt), including (100), (110), (104), (114), (212), and (216), indicating that AFt was successfully formed.

#### 2.2.2. Modification of Ettringite

The AH and AFt were added to a mixed solution of deionized water and anhydrous ethanol, followed by continuous stirring at room temperature for 30 min to obtain a uniformly dispersed solution A. AG was slowly added dropwise into an ethanol/deionized water mixed solution and stirred continuously at 25 °C for 0.5 h to ensure complete hydrolysis, yielding a hydrolysate [31]. Subsequently, the hydrolyzed AG solution was slowly added to solution A. Then, the resulting mixture was stirred continuously at 50 °C for 4 h to ensure sufficient reaction. After the reaction was complete, the precipitate was collected by centrifugation at a speed of 10,000 rpm for 10 min at 15 °C and thoroughly washed three times with anhydrous ethanol to remove the residual unreacted AG solution. The obtained product was dried under vacuum at 40 °C for 12 h, then ground and sieved to obtain AFt modified by AH and AG (AH-AFt).

#### 2.2.3. Preparation of Polymer Cement-Based Coatings

**Figure 2:**
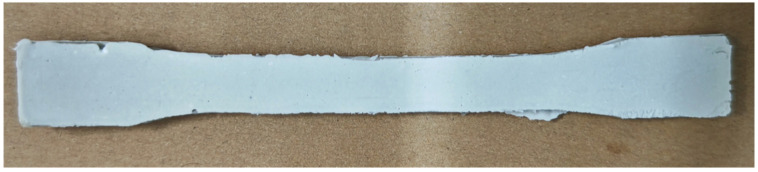
Photographs of the prepared PCC samples.

The PCC were prepared based on the mix proportions presented in Table 3 and photographs of the prepared samples are shown in Figure 2. First, the powdered materials were introduced into a mixing drum and dry-mixed for 30 min to ensure uniform dispersion. Subsequently, the obtained dry-mixed materials were sealed and stored in a drying oven for subsequent use. Meanwhile, the emulsion, water, and defoamer were mixed to prepare a solution and stirred for 30 s to obtain a homogeneous solution. Finally, the prepared solution was mixed with the pre-mixed powder in the mixer. The mixture was stirred in a mixer at 300 rpm for 2 min, followed by stirring at 800 rpm for 1 min.

### 2.3. Test Methods

Tensile strength and elongation were determined in accordance with ASTM D638-22 [32], while bond strength was measured following GB/T 16777-2008 [33]. For each group, six parallel specimens were tested for tensile strength, elongation, and bond strength measurements, and the reported values represent the average of the six measurements. The obtained slurry was cast into the corresponding molds and cured under standard conditions at a temperature of (23 ± 2) °C and a relative humidity of (50 ± 10)%. After curing to the specified age, the back side of the specimens was placed upward and dried in an oven at (40 ± 2) °C for 48 h. The specimens were then removed and cooled to room temperature in a desiccator prior to testing. The tensile specimens were prepared according to the dimensions of Type I specimens specified in ASTM D638-22. The tensile properties and elongation at break of the hardened specimens cured for 4 d, 7 d, and 28 d were measured using an electronic universal testing machine at a loading rate of 5 mm/min. The elongation was calculated according to Equation (1):

(1) $$EI=\frac{L-L_{0}}{L_{0}}\times 100$$

In Equation (1), *EI* was defined as the elongation at break, *L* was denoted as the gauge length at fracture, and *L*_0_ was the initial gauge length, which was 25 mm.

XRD was performed using a Bruker D8 Advance diffractometer. The chemical structure, pore structure, and nanomechanical properties of polymer cement-based coatings were characterized by FT-IR, a mercury intrusion porosimeter (MIP), and nanoindentation, respectively. The alcohol lamp burning test was employed to quantitatively evaluate the combustion behavior and self-extinguishing characteristics of the material under direct flame exposure. The limiting oxygen index (LOI), determined according to ASTM D2863-23e1 [34], was used to identify the minimum oxygen concentration required to sustain continuous combustion of the material. Hardness (H) and the elastic modulus (E_r_) were two common parameters in nanoindentation. The corresponding formulas were given as follows.

(2) $$H=\frac{P_{max}}{A_{c}}$$

(3) $$E_{r}=\frac{\sqrt{\pi }}{2\beta }\frac{S}{\sqrt{A}}$$

In Equation (2), *P_max_* was defined as the maximum applied load during indentation, and *A_C_* was denoted as the projected contact area of the hardness impression. In Equation (3), *S* was the contact stiffness derived from the initial unloading curve slope, *A* was the projected contact area under the applied load, and *β* was a constant that depends on the geometry of the indenter [35].

## 3. Results and Discussion

### 3.1. Mechanical Properties

#### 3.1.1. Tensile Strength

**Figure 3:**
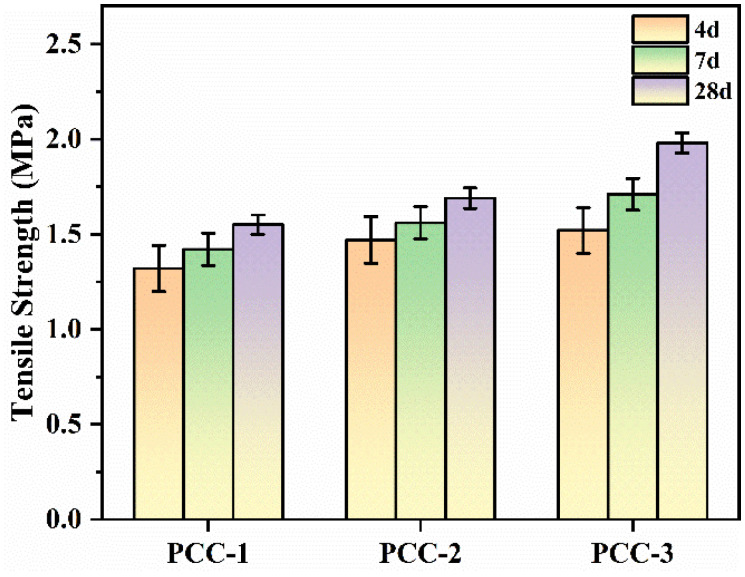
Tensile strength of PCC.

As illustrated in Figure 3, the tensile strength of PCC-3 at 4 d, 7 d, and 28 d was approximately 15.2%, 20.4%, and 27.7% higher than that of PCC-1, respectively. The tensile strength of PCC-3 at 4 d, 7 d, and 28 d was approximately 3.4%, 9.6%, and 17.2% higher than that of PCC-2, respectively. The main reason for the increase in the tensile strength of PCC-3 might be due to the interface coupling effect of AG [36]. After hydrolysis, AG generated silanol groups, which could be anchored onto the hydroxylated surface of AFt through condensation and interfacial interactions, thus forming a more stable modified inorganic surface. Meanwhile, the amino-functional organic end of AG improved the compatibility between the modified AFt and the VAE polymer phase through hydrogen bonding and polar interactions. As a result, the dispersion of AFt in the polymer–cement matrix was improved, the organic–inorganic interfacial adhesion was strengthened, and the stress transfer efficiency was enhanced, thereby reducing interfacial debonding and improving the mechanical performance of PCC [37,38,39]. This interface coupling promotes the formation of a more continuous polymer inorganic network, reduces the interface debonding under tensile load, and significantly improves the tensile strength.

#### 3.1.2. Elongation

**Figure 4:**
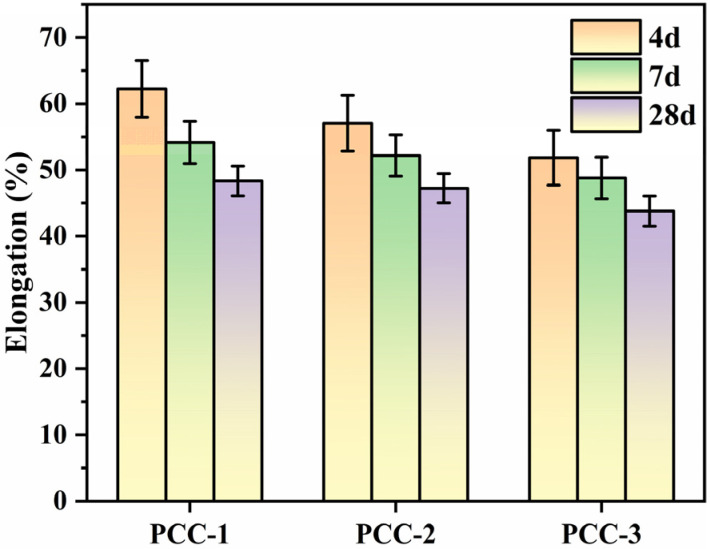
Elongation of PCC.

As illustrated in Figure 4, the elongation of all groups decreased with increasing curing age, and the values consistently followed the order of PCC-1 > PCC-2 > PCC-3 throughout the testing period. The elongation of PCC-3 at 4 d, 7 d, and 28 d was approximately 16.7%, 9.9%, and 9.4% lower than that of PCC-1, respectively. The elongation of PCC-3 at 4 d, 7 d, and 28 d was approximately 9.1%, 6.5%, and 7.3% lower than that of PCC-2, respectively. This indicated a gradual transition from a more ductile composite structure to a harder composite structure. The elongation of PCC-3 was decreased significantly, which might be due to the strong interface coupling between modified AFt and VAE polymer induced by silane coupling agent. The mobility and deformation ability of the polymer chains were restricted by the enhanced interfacial interaction, resulting in lower plasticity but higher tensile strength. With the progress of curing, the matrix became more compact, and the binding effect of the strengthened interface was more pronounced, leading to a further reduction in elongation [40].

#### 3.1.3. Bond Strength

**Figure 5:**
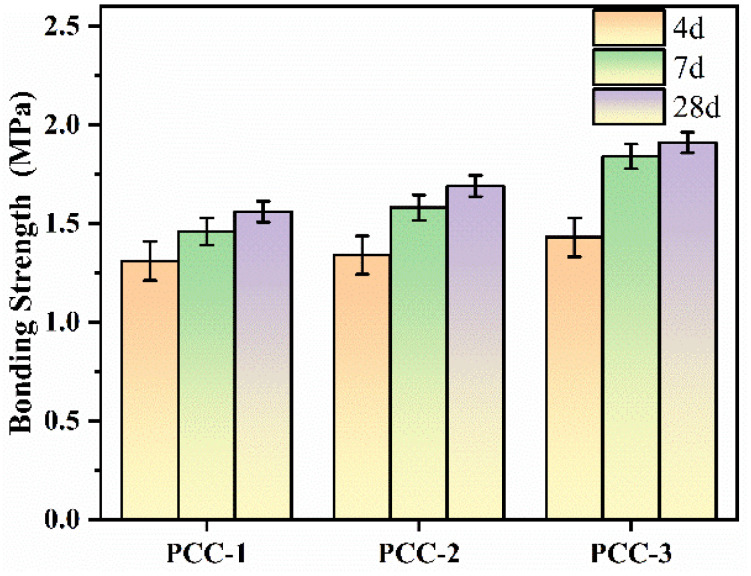
Bonding strength of PCC.

Unlike the tensile strength shown in Figure 2, bond strength reflects the resistance of the coating/substrate interface to debonding and is therefore an important indicator of the adhesion performance and practical reliability of the coating in service. In this study, the bond strength test was carried out on a cement mortar substrate according to GB/T 16777-2008. As shown in Figure 5, the bond strength of PCC-3 was significantly higher than that of PCC-1 and PCC-2. The bond strength of PCC-3 at 4 d, 7 d, and 28 d was approximately 9.2%, 26.0%, and 22.4% higher than that of PCC-1, respectively. Meanwhile, the bond strength of PCC-3 at 4 d, 7 d, and 28 d was approximately 6.7%, 16.5%, and 13.0% higher than that of PCC-2, respectively. This may be attributed to the hydrolysis of AG into silanol groups, which could bond with the surfaces of inorganic phases such as AFt through condensation and interfacial interactions. The modified AFt was then more effectively incorporated into the polymer phase, leading to a more stable organic–inorganic interface. Meanwhile, bonds were formed between the organic functional groups in AG and the polymer phase through hydrogen bonds, thereby enhancing the compatibility of the organic–inorganic interface, promoting the integrity of the composite structure, and consequently improving the adhesion performance of the coatings [41]. Therefore, in the middle and late stages, the bond strength of PCC-3 remains significantly superior to that of other samples.

### 3.2. FT-IR and XRD Analysis

**Figure 6:**
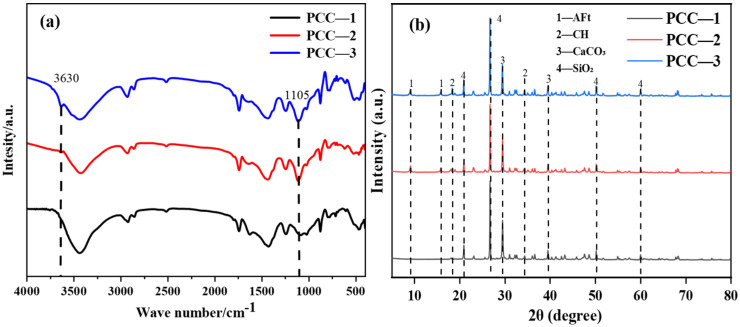
Structural characterization of PCC: (**a**) FT-IR, (**b**) XRD.

The FT-IR spectrum of PCC was presented in Figure 6a. The absorption band at approximately 1105 cm^−1^ was observed in PCC-2 and PCC-3, whereas this band was absent in PCC-1. This peak was attributed to the stretching vibration of S–O bonds, which was derived from the introduction of AFt and AH-AFt phases in PCC-2 and PCC-3, thereby confirming the successful incorporation of sulfate-containing hydration products into the coatings matrix [42]. In PCC-3, a distinct and sharp absorption peak was observed at approximately 3630 cm^−1^, which can be attributed to the enhanced O–H vibration. This indicates that the vibrations caused by the hydroxyl groups of the introduced AH molecules were significantly strengthened in PCC-3 [43]. As shown in Figure 6b, the characteristic diffraction peaks of AFt appeared at approximately 9.1° and 15.8°. Additionally, the peak positions of AFt and AH–AFt were the same, and no new characteristic peaks were detected, indicating that the modification treatment did not alter the crystal structure of AFt [3,44]. The unchanged crystal structure was considered to suggest that the modification process mainly occurred on the surface of AFt rather than within its crystal framework. This was important because the crystalline structure of AFt was closely related to its chemically bound water, thermal decomposition behavior, and functional role in the present system. Therefore, the preservation of the crystal structure of AFt was considered beneficial for maintaining its original flame-retardant potential, while the surface modification was found to improve its interfacial compatibility with the matrix.

### 3.3. MIP Analysis

**Figure 7:**
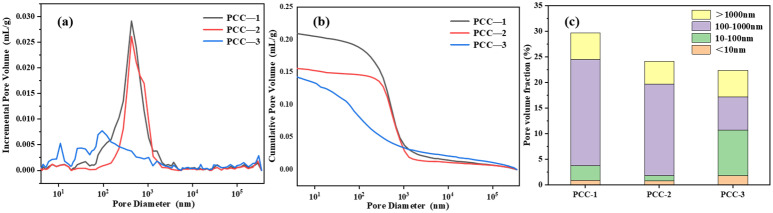
Pore structure characteristics of PCC: (**a**) Pore size distribution (**b**) Cumulative porosity (**c**) Incremental pore volume.

As shown in Figure 7, the total porosity of PCC-1, PCC-2, and PCC-3 was 29.70%, 24.13%, and 22.38%, respectively, and exhibited a gradual decrease. Among them, the total porosity of PCC-3 was reduced by 24.65% compared with PCC-1, indicating that a more compact pore structure was formed. The pores with diameters in the range of 100–1000 nm were found to account for 74.29% and 69.87% of the total pores in PCC-1 and PCC-2, respectively. Notably, the proportion of pores within this diameter range in PCC-3 was significantly decreased to only 29.01%, which was 45.28% and 40.86% lower than that of PCC-1 and PCC-2, respectively. These results indicate that the incorporation of modified AFt effectively refined the pore structure and reduced the proportion of large pores [45,46]. This pore refinement can be attributed to the improved dispersion of AH-AFt and the enhanced interfacial compatibility between the modified filler and the polymer–cement matrix. The strengthened interfacial interaction helped reduce local agglomeration and interfacial defects, thereby promoting the formation of a denser and more continuous internal structure [47].

### 3.4. Flame Retardancy Test

**Figure 8:**
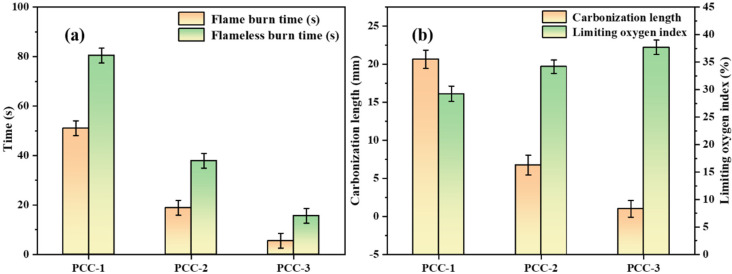
Flame retardancy of PCC: (**a**) Flame burn time and Flameless burn time (**b**) Carbonization length and Limiting oxygen index.

The test results of alcohol lamp vertical combustion and limiting oxygen index (LOI) showed that different samples had significant effects on the flame-retardant properties of cement coatings. As shown in Figure 8a, the flame burn time of PCC-1, PCC-2, and PCC-3 was measured as 51.05 s, 18.85 s, and 5.58 s, respectively. Compared with PCC-1, the flame burn time of PCC-2 and PCC-3 was reduced by approximately 63.1% and 89.1%, respectively. The flameless burn time of PCC-1, PCC-2, and PCC-3 was measured as 80.44 s, 37.91 s, and 15.64 s, respectively. The flameless burn time of PCC-2 and PCC-3 was reduced by 52.9% and 80.6%, respectively, compared with PCC-1. In conclusion, the continuous combustion behavior of the coating was significantly inhibited by the introduction of AH–AFt. The flame burn time and the flameless burn time were markedly shortened, thereby enhancing the flame retardancy of the material. In terms of the degree of flame damage, the carbonization lengths of PCC-1, PCC-2, and PCC-3 were 20.5 mm, 6.7 mm, and 2.0 mm respectively. Compared with PCC-1, the carbonization lengths of PCC-2 and PCC-3 were reduced by approximately 67.3% and 90.2%, respectively. This indicated that the flame propagation range and the degree of thermal damage were effectively inhibited. This might also have been related to the formation of a more continuous inorganic residual structure during the combustion process. This structuracted as a protective barrier, preventing heat transfer, oxygen diffusion, and flame propagation, thereby suppressing the carbonization expansion.

As shown in Figure 8b, the LOI of PCC-1, PCC-2, and PCC-3 were measured as 29.23%, 34.17%, and 37.68%, respectively. The LOI of PCC-3 was increased by 28.91% and 10.27% compared with PCC-1 and PCC-2, respectively. This indicated that PCC-3 was more difficult to maintain under continuous combustion in air. The improvement of flame-retardant performance in this study is mainly due to the dehydration and endothermic effect of AFt and Al (OH)_3_ in the heating process, which effectively reduces the thermal feedback in the combustion process and shortens the combustion duration. Silane coupling agent itself does not participate in the flame-retardant reaction, but makes the dehydration and endothermic process more uniform and effective by improving the dispersion of AFt in the coatings system and its interface with the matrix. The continuous inorganic residual structure formed further inhibited flame propagation and carbonization expansion; thus, the structure exhibited a synergistic effect on the macro flame-retardant index.

### 3.5. Nanoindentation

**Figure 9:**
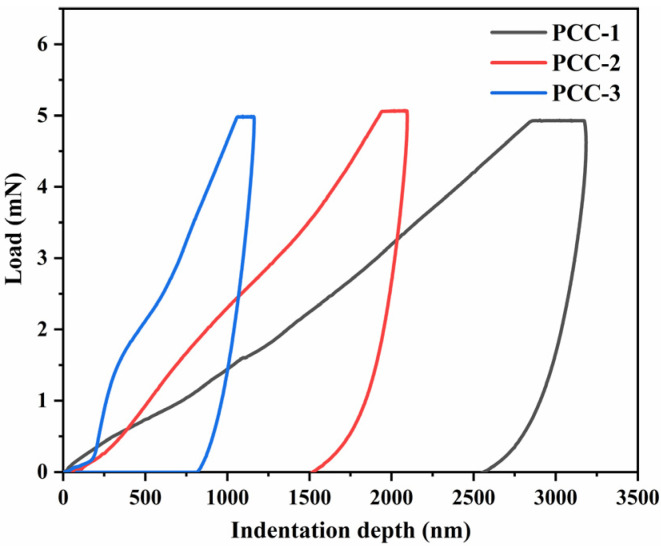
Nanoindentation load–displacement curves of PCC samples.

As shown in Figure 9, the nanoindentation load–displacement curves indicate that the micro-mechanical responses of these three groups of samples gradually show an upward trend from PCC-1 to PCC-3. Under the same load, the maximum indentation depths of PCC-1, PCC-2, and PCC-3 were measured to be 3166 nm, 2078 nm, and 1159 nm, respectively. The indentation hardness of PCC-1, PCC-2, and PCC-3 was 0.022 GPa, 0.055 GPa, and 0.194 GPa, respectively. Meanwhile, the elastic moduli of PCC-1, PCC-2, and PCC-3 were measured to be 1.42 GPa, 2.47 GPa, and 5.25 GPa, respectively. Compared with PCC-1 and PCC-2, the indentation hardness of PCC-3 was increased by approximately 781.8% and 252.7%, respectively, and its elastic modulus was increased by approximately 269.7% and 112.6%, respectively. The amphiphilic groups acted as molecular bridges between AFt and the vinyl ether polymer, enabling AG to function as an effective interfacial binder that strengthened organic–inorganic interfacial coupling [32]. This enhanced interfacial continuity suppressed debonding under localized loading, thereby accounting for the significantly improved nanomechanical response and tensile strength of the composite [28,34].

## 4. Conclusions

This study investigated the influence of amphiphilic silane-AH-AFt on the mechanical performance and flame retardancy of polymer cement-based composite coatings. The main conclusions are summarized as follows:

(1) The incorporation of AH-AFt significantly enhanced the mechanical properties. Compared with PCC-1, PCC-3 exhibited increases of up to 27.7% in tensile strength and 22.4% in bond strength at 28 d.

(2) AH-AFt refined the pore structure and reduced total porosity, contributing to a denser microstructure. The total porosity of PCC-3 was reduced by 24.65% compared with PCC-1.

(3) Nanoindentation results confirmed improved local stiffness and hardness, indicating effective interfacial strengthening induced by silane modification. The indentation hardness of PCC-3 increased by 781.8% compared with PCC-1, and the elastic modulus increased by 269.7% compared with PCC-1.

(4) The flame-retardant performance was markedly improved. PCC-3 showed substantial reductions in burning duration, flameless time, and carbonization length, while the limiting oxygen index increased from 29.23% to 37.68%.

Overall, AH-AFt serves as an efficient multifunctional inorganic filler, enabling polymer–cement coatings to achieve both enhanced mechanical reliability and superior fire resistance, with promising potential for underground protective applications.

## Figures and Tables

**Table 1:** Comparison of flame retardant types and their mechanisms.

Flame Retardant Type	Representative Systems	Flame Retardant Mechanism	Advantages	Limitations	Recent Developments
Halogenated flame retardants	Brominated FRs (PBDEs), chlorinated paraffins	Gas-phase radical quenching by releasing HCl or HBr that reacts with H and OH radicals	High flame retardant efficiency; low loading required; cost-effective	Toxic and corrosive gases during combustion; environmental persistence; bioaccumulation; regulatory restrictions	Gradual reduction of use due to environmental regulations; replacement by halogen-free systems
Non-halogenated flame retardants	Phosphorus-based (DOPO derivatives), nitrogen-based systems, silicon-based additives, inorganic fillers (Al(OH)_3_, Mg(OH)_2_)	Condensed-phase char formation, endothermic decomposition, barrier formation, smoke suppression	Low toxicity; environmentally friendly; reduced smoke generation; multifunctionality	Often require higher loading; possible influence on mechanical properties	Development of bio-based FRs, nanomaterial-reinforced FR systems, and hybrid organic-inorganic flame-retardant coatings

**Table 2:** Chemical composition of WPC (wt.%).

Component	CaO	SiO_2_	SO_3_	MgO	Al_2_O_3_	K_2_O	Na_2_O	Others	Loss
Content	65.10	18.81	1.85	0.64	7.73	0.61	0.41	4.23	0.62

**Table 3:** Composition of polymer cement-based coatings (wt.%).

Sample	VAE	Defoamer	Water	WPC	QP	CaCO_3_	AFt	AH-AFt	SP
PCC-1	30	0.45	10	25	15	20	0	0	0.1
PCC-2	30	0.45	10	25	15	10	10	0	0.1
PCC-3	30	0.45	10	25	15	10	0	10	0.1

## Data Availability

The original contributions presented in this study are included in the article. Further inquiries can be directed to the corresponding author.

## References

[1] Wang M., Wang R., Yao H., Farhan S., Zheng S., Wang Z., Du C., Jiang H. (2016). Research on the mechanism of polymer latex modified cement. Constr. Build. Mater..

[2] Liang C., Zhao P., Zou H., Song Q., Hou P., Huang Y., Wang S., Lu L. (2023). Introducing fiber to enhance the mechanical properties and durability of polymer-modified cement-based coating. Constr. Build. Mater..

[3] Wu Z., Liu J., Zhang G., Wang Y., Wang Y. (2021). Effect of aluminum sulfate alkali-free liquid accelerator with compound alkanol lamine on the hydration processes of Portland cement. Constr. Build. Mater..

[4] Moon H.Y., Shin D.G., Choi D.S. (2007). Evaluation of the durability of mortar and concrete applied with inorganic coating material and surface treatment system. Constr. Build. Mater..

[5] Jo Y.K. (2020). Adhesion in tension of polymer cement mortar by curing conditions using polymer dispersions as cement modifier. Constr. Build. Mater..

[6] Garcia T., Blanco A., Cavalaro S.H.P. (2016). Shear behaviour of sprayed concrete. Constr. Build. Mater..

[7] Wang J., Tan Y., Li H., Wang J., Gao Y. (2024). Comparative study on the moisture transfer properties of shotcrete and casting concrete. Constr. Build. Mater..

[8] Pandiyarajan N., Nunthavarawong P. (2024). Recent advancements in sealants solutions for surface coatings: A comprehensive review. J. Bio-Tribo-Corros..

[9] Lyon S.B., Bingham R., Mills D.J. (2017). Advances in corrosion protection by organic coatings: What we know and what we would like to know. Prog. Org. Coat..

[10] Pan X., Shi Z., Shi C., Ling T.C., Li N. (2017). A review on concrete surface treatment Part I: Types and mechanisms. Constr. Build. Mater..

[11] Patel R., Chaudhary M.L., Patel Y.N., Chaudhari K., Gupta R.K. (2025). Fire-Resistant Coatings: Advances in Flame-Retardant Technologies, Sustainable Approaches, and Industrial Implementation. Polymers.

[12] Nasir K.M., Sulong N.H.R., Johan M.R., Afifi A.M. (2020). Synergistic effect of industrial-and bio-fillers waterborne intumescent hybrid coatings on flame retardancy, physical and mechanical properties. Prog. Org. Coat..

[13] Liu Q., Lu Z., Hu X., Chen B., Li Z., Liang R., Sun G. (2021). A mechanical strong polymer-cement composite fabricated by in situ polymerization within the cement matrix. J. Build. Eng..

[14] Jeon K., Jeon C., Choi W. (2023). Effect of mixing ratio on fire resistance of cement mortar with polypropylene fiber and polymer. Case Stud. Constr. Mater..

[15] Han J., Liang G., Gu A., Ye J., Zhang Z., Yuan L. (2013). A novel inorganic–organic hybridized intumescent flame retardant and its super flame retarding cyanate ester resins. J. Mater. Chem. A.

[16] Hu X., Luo Y., Liu W., Sun Z. (2022). Synergistic interaction between inorganic layered materials and intumescent fire retardants for advanced fire protection. Carbon.

[17] Feng C., Liang M., Chen W., Huang J., Liu H. (2015). Flame retardancy and thermal degradation of intumescent flame retardant EVA composite with efficient charring agent. J. Anal. Appl. Pyrolysis.

[18] Li G., Wang Z., Sun W., Huang B., Tong Y., Qu J., Zhang H., Liu L., Liu M., Li S. (2025). Preparation of high-performance epoxy-based building coatings: MXene functionalized phosphorus-nitrogen curing agent synchronously improves mechanical and flame retardant performance. Constr. Build. Mater..

[19] Fan T., Yan Z., Huang W., Feng W., Bai Y., Feng C., Wu F. (2025). A comprehensive review of contents, toxic effects, metabolisms, and environmental behaviors of brominated and organophosphorus flame retardants. J. Hazard. Mater..

[20] He Z., Liu Y., Liang Y., Sun Y., Song S., Cui X. (2025). Hybrid red phosphorus/melamine microcapsules for enhanced fire resistance and PH3 suppression of polyurethane composite foam. Chem. Eng. J..

[21] Bi X., Song K., Zhang H., Pan Y.T., He J., Wang D.Y., Yang R. (2024). Dimensional change of red phosphorus into nanosheets by metal–organic frameworks with enhanced dispersion in flame retardant polyurea composites. Chem. Eng. J..

[22] Chen B., Johannes K., Ratel L., Horgnies M., Morin V., Kuznik F. (2021). Investigation on ettringite as a low-cost high-density thermochemical heat storage material: Thermodynamics and kinetics. Sol. Energy Mater. Sol. Cells.

[23] Zhou Q., Glasser F.P. (2001). Thermal stability and decomposition mechanisms of ettringite at <120 °C. Cem. Concr. Res..

[24] Zhang L., Zheng H., Xie H. (2023). Molecular Dynamics Study on Interfacial Strengthening Mechanisms of Ettringite/Polymer Nanocomposites. Buildings.

[25] Wen O.Y., Tohir M.Z.M., Yeaw T.C.S., Razak M.A., Zainuddin H.S., Hamid M.R.A. (2023). Fire-resistant and flame-retardant surface finishing of polymers and textiles: A state-of-the-art review. Prog. Org. Coat..

[26] Xie Y., Hill C.A., Xiao Z., Militz H., Mai C. (2010). Silane coupling agents used for natural fiber/polymer composites: A review. Compos. Part A Appl. Sci. Manuf..

[27] Oh M.J., Kownacki I., Ortyl J. (2025). “Silatranization”: Surface modification with silatrane coupling agents. Adv. Colloid Interface Sci..

[28] Li S., Duan Y., Zheng H., Hou D., Sui S., Liu A., Wang P. (2023). Adhesion performance of ettringite at the interface with silane and GO/silane: Insights into molecular dynamics simulations. ACS Omega.

[29] Cao W., Zhu H. (2024). A Study on the Application Performance of High-Aspect-Ratio Nano-Ettringite in Photocurable Resin Composites. Materials.

[30] Fan C., Wang B., Xu Y. (2023). Solidification/stabilization and immobilization mechanism of Pb (II) and Zn (II) in ettringite. Cem. Concr. Res..

[31] Han X., Cao Z., Wang R., He P., Zhang Y., Yu J., Ge Y. (2020). Effect of silane coupling agent modified zeolite warm mix additives on properties of asphalt. Constr. Build. Mater..

[32] (2022). Standard Test Method for Tensile Properties of Plastics.

[33] (2008). Test Methods for Building Waterproofing Coatings.

[34] (2023). Standard Test Method for Measuring the Minimum Oxygen Concentration to Support Candle-Like Combustion of Plastics (Oxygen Index).

[35] Pacaphol K., Aht-Ong D. (2017). The influences of silanes on interfacial adhesion and surface properties of nanocellulose film coating on glass and aluminum substrates. Surf. Coat. Technol..

[36] Aziz T., Ullah A., Fan H., Jamil M.I., Khan F.U., Ullah R., Iqbal M., Ali A., Ullah B. (2021). Recent progress in silane coupling agent with its emerging applications. J. Polym. Environ..

[37] Chen B., Shao H., Li B., Li Z. (2020). Influence of silane on hydration characteristics and mechanical properties of cement paste. Cem. Concr. Compos..

[38] Ma H., Zong M., Liu H., Chen C., Zhu P. (2025). Enhancement mechanism of hydrophobicity and mechanical properties of gypsum based mortar by KH550 functionalized nano mineral composite coating. Constr. Build. Mater..

[39] Feng H., Le H.T.N., Wang S., Zhang M.H. (2016). Effects of silanes and silane derivatives on cement hydration and mechanical properties of mortars. Constr. Build. Mater..

[40] Karna N., Joshi G.M., Mhaske S.T. (2023). Structure-property relationship of silane-modified polyurethane: A review. Prog. Org. Coat..

[41] Pape P.G. (2011). Adhesion promoters: Silane coupling agents. Applied Plastics Engineering Handbook.

[42] Gastaldi D., Canonico F., Boccaleri E. (2009). Ettringite and calcium sulfoaluminate cement: Investigation of water content by near-infrared spectroscopy. J. Mater. Sci..

[43] Scholtzová E., Kucková L., Kožíšek J., Tunega D. (2015). Structural and spectroscopic characterization of ettringite mineral–combined DFT and experimental study. J. Mol. Struct..

[44] Song W., Guo T., Han P., Wang X., Ma F., He B. (2023). Durability study and mechanism analysis of red mud-coal metakaolin geopolymer concrete under a sulfate environment. Constr. Build. Mater..

[45] Deng X., Li M., Wang Y., Wang J., Zhang J., Yang Z., He X., Yang J., Tan H. (2024). Impact of ettringite seeding on hydration, strength and shrinkage of Na2SO4 activated slag. Compos. Part B Eng..

[46] Wang H., Feng P., Liu X., Shi J., Wang C., Wang W., Li H., Hong J. (2024). The role of ettringite seeds in enhancing the ultra-early age strength of Portland cement containing aluminum sulfate accelerator. Compos. Part B Eng..

[47] Feng B., Liu J., Chen Y., Tan X., Zhang M., Sun Z. (2022). Properties and microstructure of self-waterproof metakaolin geopolymer with silane coupling agents. Constr. Build. Mater..

